# Effect of Early Expressed Human Milk on Insulin-Like Growth Factor 1 and Short-Term Outcomes in Preterm Infants

**DOI:** 10.1371/journal.pone.0168139

**Published:** 2016-12-14

**Authors:** Francesca Serrao, Patrizia Papacci, Simonetta Costa, Carmen Giannantonio, Francesco Cota, Giovanni Vento, Costantino Romagnoli

**Affiliations:** Department of Pediatrics, Division of Neonatology, Catholic University of the Sacred Heart, Rome, Italy; Centre Hospitalier Universitaire Vaudois, FRANCE

## Abstract

**Aims:**

Preterm breast milk contains high levels of bioactive components, including insulin-like growth factor 1 (IGF-1), that are reduced by Holder pasteurization. Animal studies have shown that milk-borne IGF-1 is likely absorbed intact in a bioactive form by the intestines. The aim of this study was to assess if early non-pasteurized expressed breast milk nutrition may affect IGF-1 plasma levels in premature infants. We also investigated the possible association between early expressed milk nutrition and short-term outcomes.

**Methods:**

Fifty-two preterm infants with gestational age < 31 weeks were divided into two groups according to expressed breast milk intake (< or ≥ 50 mL/Kg/day) until 32 weeks of postmenstrual age when blood sampling for IGF-1 analysis was performed.

**Results:**

In our population, early expressed breast milk does not affect IGF-1 plasma levels (*p* 0.48). An association was observed between early expressed milk nutrition and a lower incidence of bronchopulmonary dysplasia, sepsis, feeding intolerance, need for parenteral nutrition and length of hospitalization.

**Conclusions:**

Contrary to the results in some animal studies, our results did not seem to show that early expressed breast milk can help to maintain postnatal IGF-1 near foetal levels in preterm infants. The observed protective effect of expressed breast milk on short-term outcomes can be the starting point for further study of the effects of non-pasteurized human milk in preterm infants.

## Introduction

Human milk should be recommended for premature infants, either by direct breastfeeding and/or using the mother’s own expressed milk [1]. Human milk can be pasteurized to avoid the potential transmission of infectious agents, typically by heating to 65.5°C for 30 minutes (Holder pasteurization). Some studies on Holder pasteurization, however, have shown the reduction or complete inactivation of bioactive components such as immunological, growth and anti-oxidant factors. These components are present in higher concentrations in the milk of mothers of preterm infants and are especially important during the early postnatal phase [2–3]. In particular, the premature breast milk contains high concentrations of insulin-like growth factor 1 (IGF-1) that are reduced by approximately 40% upon Holder pasteurization [4–5].

IGF-I is a 7.5-kDa single chain peptide that plays crucial roles mainly in foetal growth, retinal vascularization and maturation of the developing brain.

Foetal IGF-1 levels are regulated by foeto-maternal placental interaction. Using cordocentesis normal values of foetal plasma IGF-1 were determined according to gestational age [6]. They decrease rapidly at birth due to low endogenous production, and very preterm infants continue to have low levels for several weeks. IGF-1 plasma levels are positively correlated with gestational age, postnatal age and nutritional intake [7]; however, recent studies have shown that during the early postnatal phase, adequate nutritional intake is not enough to bring IGF-1 to as high as foetal levels, suggesting immaturity of the endogenous production. IGF-1 endogenous production begins at 32 weeks postmenstrual age (PMA) [8]. For this reason, exogenous IGF-1 administration is a focus of on-going studies. Milk-borne IGF-1 acts as a growth factor for gut maturation; however, some animal studies have shown possible absorption into the circulation with a dose-dependent increase in orally administered IGF-1 and protective action of milk proteins on IGF-1 degradation [9–14]. The aim of this study was to assess whether, in premature infants, early non-pasteurized expressed breast milk (EBM) nutrition might affect IGF-1 plasma levels. We also investigated the possible association between early EBM nutrition and short-term clinical outcomes such as bronchopulmonary dysplasia (BPD), sepsis, feeding tolerance, need for parenteral nutrition, retinopathy of prematurity (ROP) and postnatal growth.

## Materials and Methods

### Study population

The study was a prospective, longitudinal, observational cohort study that included all preterm infants born at gestational age < 31 weeks admitted into our neonatal intensive care unit for whom written informed consent was obtained from the parents. Informed consent was included in the medical record. Exclusion criteria were common conditions that influence IGF-1 plasma levels: intrauterine growth restriction, small for gestational age, insulin or steroid therapy; moreover, infants with cerebral lesions or any conspicuous congenital anomaly were excluded. The Ethical Committee of Gemelli University Hospital approved the study. The study population was divided into the following two groups according to the EBM intake during the early postnatal period until 32 weeks PMA: infants who consumed mean EBM ≥ 50 mL/Kg/day (group A) and infants who consumed mean EBM <50 mL/Kg/day (group B).

### Nutritional protocol

Parenteral nutrition was begun on the first day of life in all subjects with a birth weight less than 1250 g or less than 1500 g with severe respiratory distress. Fluid intake was begun at 60 mL/Kg/day and subsequently modulated to obtain a daily weight decrease between 2% and 5% and natraemia between 130 and 150 mEq/L.

The quantity of nutrients during the first day included glucose (6 g/Kg), amino acids (2 g/Kg), lipids (0.5 g/Kg) and calcium (40 mg/Kg), with the subsequent introduction of magnesium, phosphorus and electrolytes (3rd day of life) and oligoelements and vitamins (5th day of life).

The nutritional mixture was infused with the objective of achieving an optimum intake of 110–120 calories/Kg/day and 3.5–4 g/Kg/day of amino acids [15].

Parenteral nutrition was discontinued at a parenteral intake < 50 mL/Kg and growth rate > 15 g/Kg/day for at least 72 hours.

Sterile milk expression has been supported since the first day of life by nursing exerts and neonatologists through oral, written and iconographic information.

The milk was stored at 4°C in a dedicated refrigerator whose thermal stability was monitored periodically and was always administered within 24 hours from collection.

Before administration, the milk containers were shaken to homogenize any deposits, and the required amount was aseptically removed.

Fresh breast milk was not administered to infants born to mothers with HIV, HBV, HCV, CMV, typhoid, paratyphoid, brucellosis, pertussis, active pulmonary tuberculosis, or syphilis, or taking medication or drugs incompatible with breastfeeding and was temporarily interrupted in the case of mastitis, nipple mycosis, breast or chest herpes simplex or varicella zoster infections.

In the absence of own mother's milk, infants received pasteurized human milk for 15 days of life followed by premature formula milk.

The enteral feeding was initiated as early as possible as minimal enteral feeding (<20 mL/Kg) for at least 5 days with successive increases of 20 mL/Kg/day to ensure an adequate proportion of calories (120-130/Kg/day) and protein (3.5–4.5 g/Kg/day).

To evaluate feeding tolerance, the daily gastric residual volume and the daily episodes of emesis were recorded. The criteria for the reduction of enteral feeding were a gastric residual volume more than 4 mL/Kg after a single meal or more than 2 mL/Kg after 3 consecutive meals, or more than 3 consecutive episodes of vomiting. The criteria for complete withdrawal of enteral feeding were a gastric residual volume more than 5 mL/Kg after a single meal, abdominal distension with an increase in abdominal circumference greater than 2 cm in 24 hours, metabolic acidosis with pH below 7.20 for more than 2 hours, hypoxia with paO_2_ < 50 mm/Hg for more than 2 hours, or hypotension.

Full enteral feeding was defined by an intake of 150 mL/kg/day. Total parenteral and enteral caloric and protein intakes were calculated. Despite the variability of macronutrients, given that breast milk was also administered over the first two weeks of life, it was assumed that the raw or pasteurized human milk energy and protein content were 78 kcal/100 mL and 2.2 g/100 mL, respectively [16]. The formula used for premature infants instead contains an energy intake of 82 Kcal/100 mL and a protein intake of 2.9 g/100 mL.

Breast milk was fortified when the enteral intake reached 100 mL/kg/day or from 14 days of life.

The fortification consisted of Milupa Aptamil BMF was used, increasing the energy content by 15 Kcal/100 mL and the protein content by 1.1 g/100 mL. The amount and type of milk were recorded daily.

### Blood sampling and quantitative analysis of IGF-1

Blood sampling for IGF-1 analysis was performed at 32 weeks PMA.

After centrifugation, serum samples were stored at -80°C until assayed. The IGF-1 samples were diluted 1:21, and the IGF-1 concentrations were analysed using IGFBP-blocked ELISA IGF-1 E2 (Mediagnost, Reutlingen, Germany). The analytical sensitivity of the ELISA E20 yielded 0.09 ng/mL. The Inter- and Intra-Assay variation coefficients were less than 6.8% and 6.7%, respectively. All samples were analysed within the same assay. Laboratory analysis was blinded to the infant groups.

### Monitoring and definitions

Gestational age was determined by the best obstetric estimate based on the first day of the last menstrual period, prenatal ultrasound, and postnatal physical examination. Weight was measured daily by nursing personnel using digital scales accurate to 5 grams. Additional anthropometric measurements were performed weekly using standardized procedures. Length was measured using a fixed headboard and movable footboard, and head circumference, at the maximal occipitofrontal circumference, was measured using a non-stretchable tape accurate to the nearest millimetre. Weight, length and head circumference were compared with intrauterine reference values using Z-scores [17]. Growth performance was measured by calculating the Z-score change from birth to discharge. Values of Z-score < -1.28 for weight, length and head circumference identified small for gestational age (SGA) infants.

Ophthalmologic monitoring was performed in neonates with gestational age < 29 weeks from 29 weeks of PMA and in neonates with gestational age > 29 weeks from the second week of life, then weekly until the full vascularization of the peripheral retina.

The findings were classified into the different stages of retinopathy according to the International Classification of ROP [18].

Surgical treatment of laser photoablation or experimental therapy with antiangiogenic drugs was performed in the case of ROP zone 2, stage II or III with plus disease, in the case of zone 1 ROP with plus disease at any stage, and in the case of zone 1 ROP, stage III with or without plus disease. BPD was defined according to the guidelines [19]. Late onset sepsis (> 72 hours of life) was defined based on compatible clinical signs and symptoms and altered inflammatory markers with or without positive blood culture [20]. The necrotizing enterocolitis was defined according to the criteria of Bell [21].

### Statistical analyses

The continuous data are reported as the mean ± standard deviation or median (range); binary data are reported as count and percentage. The study population was divided into two groups according to the EBM intake during the early postnatal period until 32 weeks PMA. The Shapiro-Francia W' test was used to assess the distribution of the continuous variables. The differences between groups were evaluated by the Wilcoxon rank sum test (Mann Whitney U test) or Student's t-test as appropriate according to whether the distribution of the continuous variable was non-parametric or parametric; categorical data were analysed using Fisher's exact test. The correlation between the variables was investigated by Pearson's correlation for continuous variables (r for significant associated variables have been reported). A two-tailed p <0.05 was considered significant. Analyses were performed using Stata/IC 13.

## Results

**Fig 1:**
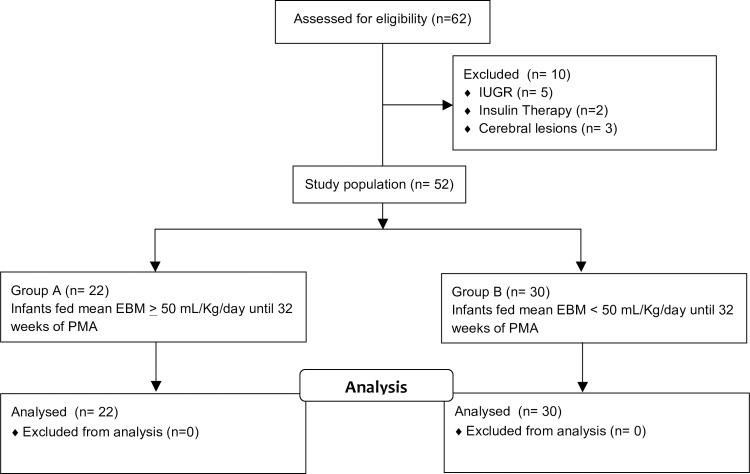
Distribution of study population

Sixty-two infants were enrolled in the study; five infants were excluded because of intrauterine growth restriction, three infants for cerebral lesions and two for an early need for insulin therapy, leaving 52 eligible infants for evaluation. The median gestational age was 28.4 weeks (range 24.9–30.7); median birth weight was 1145 g (range 510–1800). The study population was divided into two groups according to the EBM intake during the early postnatal period until 32 weeks PMA: infants who consumed mean EBM ≥ 50 mL/Kg/day (group A—22 infants) and infants who consumed mean EBM <50 mL/Kg/day (group B—30 infants) (Fig 1).

No difference was found in the baseline characteristics between the two study groups (Table 1).

**Table 1:** Baseline characteristics of preterm infants in the two study groups.

	Group A N 22	Group B N 30	*p*
Gestational age (weeks)	28.5 ± 1.5	28.3 ± 1.8	*0*.*644*
Birth weight (grams)	1150 ± 237	1140 ± 321	*0*.*603*
Male (n)	10 (47.6)	16 (51.6)	*1*.*000*
Caesarean section (n)	17 (80.9)	23 (74.2)	*0*.*525*
1’ Apgar	7.0 ± 1.1	6.1 ± 1.9	*0*.*127*
5’ Apgar	8.5 ± 0.9	8.0 ± 1.1	*0*.*061*
Crib score	1.1 ± 1.3	1.3 ± 1.6	*0*.*763*
Days at 32 weeks PMA	25.3 ± 10.0	25.7 ± 12.7	*0*.*977*

Values are expressed as the mean ± SD or number and %. P values of 0.05 were considered to be statistically significant.

Nutritional data are shown in Table 2: the caloric and protein intake were similar in the two groups; group A had fewer days of parenteral nutrition and reduced episodes of feeding interruption, and almost all infants were discharged with any EBM.

**Table 2:** Nutritional data.

	Group A N 22	Group B N 30	*p*
Age of beginning of MEF (days)	3.3 ± 2.4	4.1 ± 2.9	*0*.*321*
EBM until 32 weeks PMA (mL/Kg/day)	71.4± 22.4	10.1± 14.9	***0*.*001***
EBM until 32 weeks PMA (%)	75 (50–100)	25 (0–90)	***0*.*002***
DM until 32 weeks PMA (%)	4 (0–23)	25 (0–100)	***0*.*010***
FM until 32 weeks PMA (%)	21(0–46)	50 (0–100)	***0*.*027***
Protein intake until 32 weeks PMA (g/Kg/day)	3.5 ± 0.3	3.6 ± 204	*0*.*191*
Caloric intake until 32 weeks PMA (Kcal/Kg/day)	105 ± 4.4	107 ± 10.7	*0*.*532*
EMB until discharge (mL/Kg/day)	82.4 ± 35.0	19.0 ± 30.9	***0*.*002***
Parenteral nutrition (days)	13.3 ± 8.2	24.7 ± 18.2	***0*.*001***
Feeding interruption (n episodes)	0.2 ± 0.4	1.1 ± 1.4	***0*.*002***
Any EBM at discharge (n)	20 (90.9)	7 (23.3)	***0*.*001***

Values are expressed as the mean ± SD, number and % or median and interquartile range. P values of 0.05 were considered to be statistically significant. MEF: minimal enteral feeding; PMA: postmenstrual age; EBM: expressed breast milk; DM: donor milk; FM: formula milk

The percentage and mean intake of enteral nutrition from EBM over time to each week PMA in the two groups were shown in S1 Fig.

**Fig 2:**
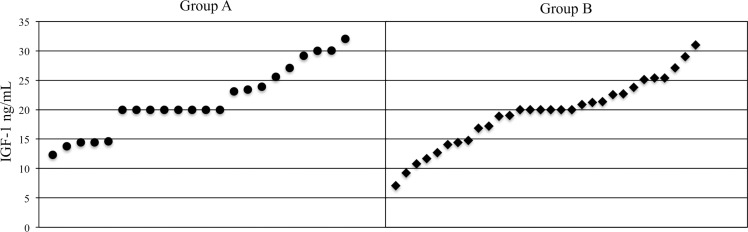
IGF-1 plasma level distribution at 32 weeks of PMA. The figure shows the individual values of IGF-1 in all newborns of group A and group B at 32 weeks of PMA: IGF-1 distribution and range values are similar in the two study groups (p 0.48).

IGF-1 was measured at a mean age of 25.4 ± 10.2 days in group A and 28.7 ± 14.0 days in group B (*p* 0.08), corresponding to 32.1 ± 0.2 weeks PMA in group A and 32.3 ± 0.4 weeks PMA in group B (p 0.41). Mean IGF-1 plasma levels were 21.5 + 5.7 ng/mL in group A and 21.2 ± 9.6 ng/mL in group B with no significant difference (p 0.48); the distribution is shown in Fig 2.

There was no correlation between the total mL EBM received and IGF-1 level (r -0,03) or between EBM as a percentage of total diet and IGF-1 levels (r 0,05).

Clinical outcomes are shown in Table 3: infants in group A had a lower incidence of BPD despite similar ventilation and oxygen therapy rates, a lower incidence of sepsis, fewer days of antibiotic therapy and a shorter length of hospital stay. There are no differences in the two groups in terms of ROP onset, ROP that required surgical treatment, or number of infants who were appropriate for gestational age at birth and became SGA at 32 weeks PMA and at discharge due to extrauterine growth restriction.

**Table 3:** Clinical outcomes.

	Group A N 22	Group B N 30	*p*
Mechanical ventilation (hours)	40.8 ± 38.9	45.2 ± 40.8	*0*.*254*
Oxygen therapy (hours)	234 ± 123	287 ± 265	*0*.*134*
BPD (%)	0 (0)	8 (26.7)	***0*.*001***
Sepsis (episodes)	0.9 ± 0.8	1.8 ± 1.7	***0*.*010***
Antibiotics (days)	10.6 ± 8.0	28.7 ± 12.1	***0*.*002***
ROP any stage (%)	11 (50.0)	19 (63.3)	*0*.*056*
Surgical ROP (%)	1 (4.5)	3 (10.0)	*0*.*431*
SGA at 32 weeks PMA (n)	2 (9.0)	4 (13.3)	*0*.*082*
SGA at discharge (n)	3 (13.6)	4 (13.3)	*0*.*145*
PMA at discharge (weeks)	35.5 ± 1.4	38.0 ± 5.6	***0*.*032***
Hospital stay (days)	51.1 ± 10.3	69.0 ± 44.7	***0*.*022***

Values are expressed as the mean ± SD or number and %. P values of 0.05 were considered to be statistically significant. BPD: bronchopulmonary dysplasia; ROP: retinopathy of prematurity; PMA: postmenstrual age; SGA: small for gestational age

## Discussion

Our objective was to test whether early EBM intake affects IGF-1 plasma levels.

This work is the first study performed on human premature infants to evaluate the intestinal absorption of expressed milk-borne IGF-1 in preterm infants.

In our study population, plasma IGF-1 levels at 32 weeks PMA were similar regardless of EBM amount, with values below foetal levels despite adequate protein intake. The 5th centile of normal foetal IGF-1 levels at 32 weeks PMA is indeed around 25 ng/mL [6]. One reason for the similar IGF-1 plasma levels in two groups could be a lack of intestinal absorption, contrary to some animal studies. However, the characteristics of the gastrointestinal tract of neonates, such as low gastric acidity and low luminal proteolytic digestion, should improve the survival of milk- borne IGF-1. Furthermore, as described in detail previously [22], in cases of extreme prematurity associated with increased intestinal permeability, biologically significant amounts of milk-borne or orally administered IGF-1 should be absorbed into the circulation. Another reason for the similar IGF-1 plasma levels in the two groups could be correlated to the amount of IGF-1 contained in EBM: despite containing greater amounts of IGF-1 than pasteurized human milk, the quantities may not be such as to ensure a significant plasma increase. Probably even if the IGF-1 in EBM is absorbed biologically intact, the “first pass” could be to the portal circulation, predominantly affecting hepatic growth and metabolism. Moreover, the IGF-1 in EBM was not dosed.

The major limitation of this study is that it is observational, but it is not feasible to perform a randomized trial of breast milk for ethical reasons. Future studies are therefore required to examine other IGF-1 administration approaches to maintain postnatal IGF-1 to near foetal levels when nutritional intake may be inadequate. In our study population of extremely low birth weight preterm infants, a mean EBM of ≥ 50 mL/Kg/day until 32 weeks of postmenstrual age was associated with a lower incidence of BPD, sepsis, feeding intolerance, need for parenteral nutrition and length of hospitalization. EBM also did not result in a greater extra-uterine growth retardation compared to the control group, suggesting that the caloric and protein intake from fortified milk after 15 days of life may be similar and that the clinical benefits favour breast milk. Some of these findings were corroborated in recent studies [23–24]. However, the observational design of our study and the population size only allow it to show an association between early EBM and short-term outcomes limited by the multifactorial pathogenesis. Our study encourages the greatest possible efforts to promote the early support of expressed breast milk in the neonatal intensive care unit and supports efforts to investigate the correlation between non-pasteurized breast milk feeding and preterm infant outcomes.

## Supporting Information

S1 Fig Percentage and mean intake of EBM over time for each week PMA. (TIF)

## References

[1] American Academy of Pediatrics: Section on Breastfeeding: Breastfeeding and the use of human milk, Pediatrics 2005; 115: 496–506. 10.1542/peds.2004-2491 15687461

[2] Ewaschuk JB, Unger S, Harvey S, O'Connor DL, Field CJ. Effect of pasteurization on selected immune components of milk: impilcation for feeding preterm infants. App Physiol Nutr Metab 2011; 33: 175–182. 10.1139/h11-008 21609278

[3] Silvestre D1, Miranda M, Muriach M, Almansa I, Jareño E, Romero FJ. Antioxidant capacity of human milk: effect of thermal conditions for the pasteurization, Acta Pædiatr 2008; 97: 1070–1074. 10.1111/j.1651-2227.2008.00870.x 18477059

[4] Goelz R, Hihn E, Hamprecht K, Dietz K, Jahn G, Poets C, Elmlinger M. Effects of different CMV-heat-inactivation-methods on growth factors in human breast milk. Pediatr Res. 2009 4; 65(4): 458–61. 10.1203/PDR.0b013e3181991f18 19127217

[5] Elmlinger MW, Hochhaus F, Loui A, Frommer KW, Obladen M, Ranke MB. Insulin-Like Growth Factors and Binding Proteins in Early Milk from Mothers of Preterm and Term Infants. Horm Res 2007; 68: 124–131. 10.1159/000100488 17341887

[6] Lassarre C, Hardouin S, Daffos F, Forestier F, Frankenne F, Binoux M. Serum insulin-like growth factors and insulin-like growth factor binding proteins in the human fetus. Relationships with growth in normal subjects and in subjects with intrauterine growth retardation. Pediatr Res 1991; 29:219–225. 10.1203/00006450-199103000-00001 1709729

[7] Engström E, Niklasson A, Albertsson Wikland K, Ewald U, Hellström A. The Role of Maternal Factors, Postnatal Nutrition, Weight Gain, and Gender in Regulation of Serum IGF-I among Preterm Infants. Pediatr Res 2005; 57: 605–610. 10.1203/01.PDR.0000155950.67503.BC 15695599

[8] Hansen-Pupp I, Löfqvist C, Polberger S, Niklasson A, Fellman V, Hellström A, Ley D. Influence of insulin-like growth factor I and nutrition during phases of postnatal growth in very preterm infants. Pediatr Res. 2011 5; 69(5 Pt 1): 448–53. 10.1203/PDR.0b013e3182115000 21263374

[9] Baumrucker CR1, Hadsell DL, Blum JW Effects of dietary insulin-like growth factor I on growth and insulin-like growth factor receptors in neonatal calf intestine. J Anim Sci. 1994 2; 72(2): 428–33. 8157526 10.2527/1994.722428x

[10] Lee CY, Head HH, Feinstein CR, Hayden J, Simmen FA: Endocrine changes and circulating insulin-like growth factors in newborn calves fed colostrums, milk or milk replacer. Asian-Aust J Anim Sci 1995; 8: 51–58.

[11] Xu RJ, Wang T: Gastrointestinal absorption of insulin-like growth factor-I in neonatal pigs. J Pediatr Gastroenterol Nutr 1996; 23: 430–437. 8956182 10.1097/00005176-199611000-00013

[12] Bastian SE, Walton PE, Ballard FJ, Belford DA. Transport of IGF-I across epithelial cell monolayers. J Endocrinol. 1999; 162(3): 361–9. 10467227 10.1677/joe.0.1620361

[13] Philipps AF, Kling PJ, Grille JG, Dvorak B. Intestinal transport of insulin-like growth factor-I (IGF-I) in the suckling rat. JPGN. 2002; 35: 539–544. 12394381 10.1097/00005176-200210000-00015

[14] Philipps AF, Dvorak B, Kling PJ, Grille JG, Koldovsky O. Absorption of milk-borne insulin-like growth factor-I into portal blood of sucklin rats. JPGN. 2000; 31: 128–135. 10941963 10.1097/00005176-200008000-00008

[15] Agostoni C, Buonocore G, Carnielli VP, De Curtis M, Darmaun D, Decsi T, et al; ESPGHAN Committee on Nutrition. Enteral nutrient supply for preterm infants: commentary from the European Society of Paediatric Gastroenterology, Hepatology and Nutrition Committee on Nutrition J Pediatr Gastroenterol Nutr. 2010 1; 50(1): 85–91. 10.1097/MPG.0b013e3181adaee0 19881390

[16] Ballard O, Morrow AL. Human milk composition: nutrients and bioactive factor. Pediatr Clin North Am. 2013 2; 60(1): 49–74. 10.1016/j.pcl.2012.10.002 23178060 PMC3586783

[17] Bertino E, Spada E, Occhi L, Coscia A, Giuliani F, Gagliardi L, et al Neonatal anthropometric chart: the Italian neonatal study compared with other European studies. J Pediatr Gastoenter Nutr 2010; 51: 353–361. 10.1097/MPG.0b013e3181da213e 20601901

[18] International Committee for the Classification of Retinopathy of Prematurity. The International Classification of Retinopathy of Prematurity revisited. Arch Ophthalmol. 2005 7; 123(7): 991–9. 10.1001/archopht.123.7.991 16009843

[19] Bancalari E, del Moral T Bronchopulmonary dysplasia and surfactant Biol Neonate. 2001 5; 80 Suppl 1:7–13. 10.1159/000047170 11359038

[20] Lim WH, Lien R, Huang YC, Chiang MC, Fu RH, Chu SM et al Prevalence and pathogen distribution of neonatal sepsis among very-low-birth-weight infants. Pediatr Neonatol. 2012; 53(4): 228–34. 10.1016/j.pedneo.2012.06.003 22964280

[21] Walsh MC, Kliegman RM. Necrotizing enterocolitis: treatment based staging criteria. Pediatr Clin North Am 1986; 33: 179–201. 3081865 10.1016/S0031-3955(16)34975-6 PMC7131118

[22] Burrin DG. Is milk-borne insulin-like growth factor-I essential for neonatal development? J Nutr. 1997 5;127(5 Suppl):975S–979S. 9164277 10.1093/jn/127.5.975S

[23] Dritsakou K, Liosis G, Valsami G, Polychronopoulos E, Skouroliakou M. Improved outcomes of feeding low birth weight infants with predominantly raw human milk versus donor banked milk and formula. J Matern Fetal Neonatal Med. 2015; 24: 1–8. 10.3109/14767058.2015.1038232 25909500

[24] Corpeleijn WE, Kouwenhoven SM, Paap MC, van Vliet I, Scheerder I, Muizer Y et al Intake of own mother's milk during the first days of life is associated with decreased morbidity and mortality in very low birth weight infants during the first 60 days of life. Neonatology. 2012;102(4):276–81. 10.1159/000341335 22922675

