# Supplementary figures and images for: Effect of Early Expressed Human Milk on Insulin-Like Growth Factor 1 and Short-Term Outcomes in Preterm Infants
Source: PLoS One. 2016 Dec 14;11(12):e0168139. doi: 10.1371/journal.pone.0168139 (PMC5156408; doi:10.1371/journal.pone.0168139)

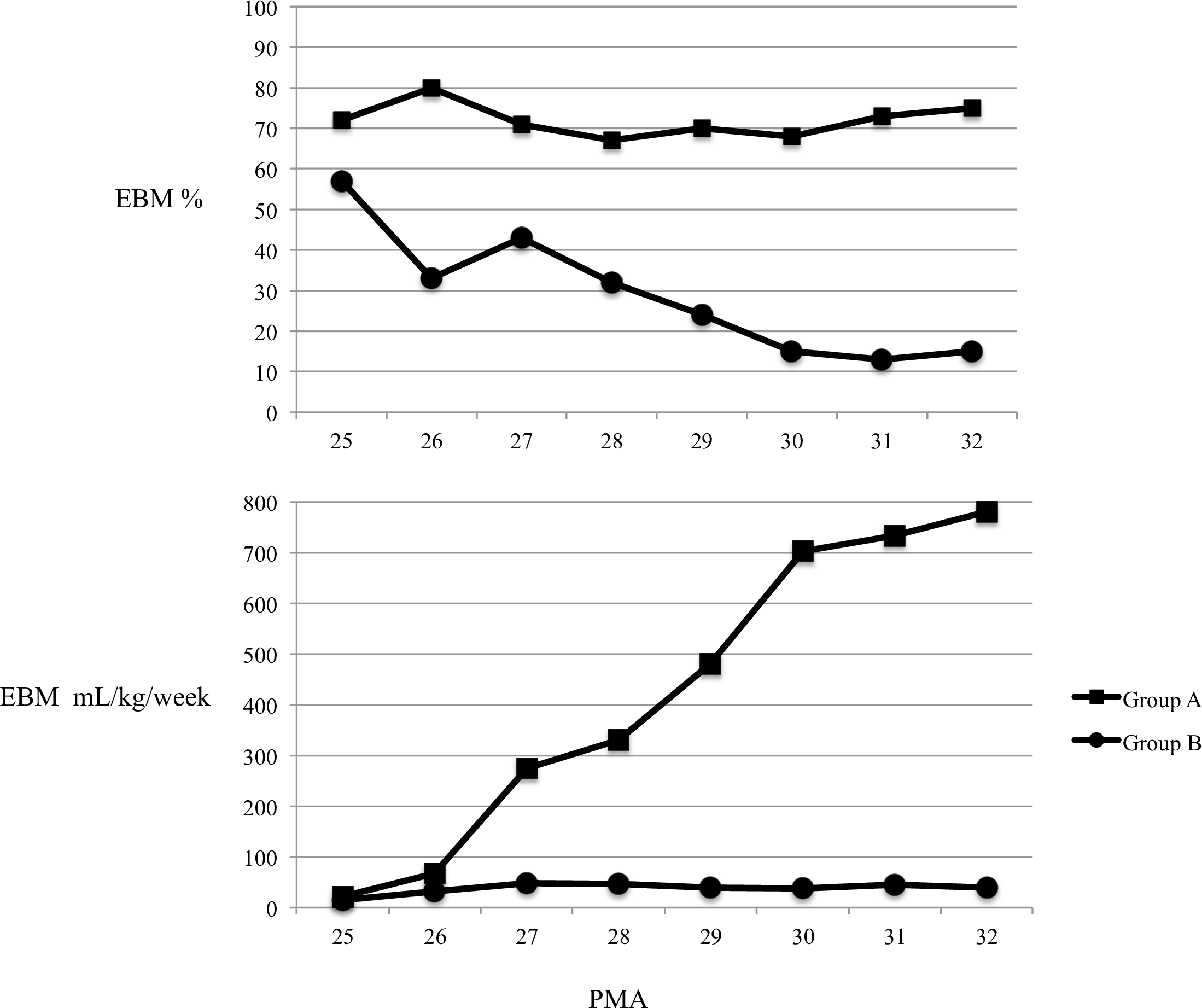

Supplement: S1 Fig — (TIF) [file pone.0168139.s001.tif]
